# MiR-206 may suppress non-small lung cancer metastasis by targeting CORO1C

**DOI:** 10.1186/s11658-020-00216-x

**Published:** 2020-03-17

**Authors:** Ming Liao, Lijun Peng

**Affiliations:** Thoracic Surgery Department, General Hospital of Southern Theater Command, PLA, No. 111, Liuhua Road, Yuexiu District, Guangzhou, 510010 China

**Keywords:** Non-small-cell lung cancer (NSCLC), microR-206 (miR-206), Proliferation, Migration and invasion, Coronin-1C (CORO1C)

## Abstract

**Object:**

Non-small lung cancer (NSCLC), with a poor 5-year survival rate (16%), is the major type of lung cancer. Metastasis has been identified as the main factor that leads to NSCLC therapy failure. MiR-206 is a metastasis suppressor in many cancers, including colorectal cancer, renal cell carcinoma and breast cancer. However, the role of miR-206 in NSCLC metastasis and the underlying mechanism are still obscure.

**Methods:**

Quantitative reverse-transcription PCR (q-RT-PCR) assay was used to detect miR-206 mRNA of NSCLC tissues and lung cancer lines. The MTT assay, scratch wound healing assay, transwell migration assay and transwell invasion assay were conducted to illuminate the effect of miR-206 on A549 cells’ proliferation, migration and invasion. Gaussia luciferase reporter assay, q-RT-PCR and western blotting assay were used to explore the underlying mechanism. Also, the A549 xenograft model was conducted to evaluate the anti-tumor effect of miR-206 in vivo.

**Results:**

The results showed that miR-206 expression was decreased in NSCLC tissues and lung cancer cells. Further research demonstrated that miR-206 inhibited the proliferation, migration and invasion of A549 cells via negatively regulating Coronin-1C (CORO1C), and CORO1C deletion significantly rescues the miR-206 mediated inhibitory effect on A549 cells. Moreover, miR-206 exhibited a perfect anti-tumor effect in the A549 xenograft model.

**Conclusion:**

Our study reveals that miR-206 functions as a tumor metastasis suppressor and sheds new light on the clinical significance of miR-206 in NSCLC therapy.

## Introduction

Lung cancer, with a morbidity of approximately 1.3 million every year, is one of the principal diseases threatening human health worldwide. Non-small-cell lung cancer (NSCLC) is the most common form of lung cancer and approximately 85% of lung cancer-related mortality was caused by NSCLC [1, 2]. Although advances in surgical therapy, chemotherapy, radiotherapy and molecular targeting therapy have been made in preclinical and clinical trials for NSCLC therapy, the overall 5-year survival rate is approximately 16% [3, 4]. Distant metastasis is the major factor that contributes to the failure of NSCLC therapy. The factors governing NSCLC metastasis are complicated, and the underlying mechanism remains unclear [5, 6]. Thus, identifying new molecules that regulate NSCLC metastasis and revealing its underlying mechanism is beneficial to promote NSCLC therapy.

Micro-RNAs (miRNAs) are 16- to 25-nucleotide noncoding RNAs that are highly conserved and are endogenous regulators of gene expression. They typically act with the 3′-untranslated region (3′-UTR) of target messenger RNAs (mRNA), inducing miRNA cleavage and then degrading the mRNA or repressing the translation process [7, 8]. MiRNAs are involved in various tumor processes, including development, metastasis, resistance and recurrence. In addition, miRNAs represent potential biomarkers and therapeutic targets for cancer [9, 10]. For example, miR-34 regulates the proliferation, survival, apoptosis and angiogenesis of many cancers. MRX34, a liposomal formulation of miR-34a, has been applied as a cancer therapy in preclinical and clinical experiments [11, 12].

Accumulating evidence has proven that many miRNAs, including miR-34a, miR-193, miR-200 and miR-204, play a key role in NSCLC metastasis [13–16]. MiR-206 is a suppressor in many cancers, such as colorectal cancer, renal cell carcinoma, and gastric cancers. Also, cell cycle arrest and suppressing epithelial mesenchymal transformation (EMT) contribute to the miR-206-mediated tumor growth inhibition effect of miR-206 [17–19]. Recently, several studies have noted that miR-206 also contributes to NSCLC metastasis, but the underlying mechanism remains unclear [20, 21].

In a word, miR-206 expression was reduced in NSCLC tissues and cells lines. The patients with low miR-206 expression exhibit a poor survival rate. Mechanism studies demonstrated that miR-206 restrained cell proliferation, migration and invasion via targeting actin-binding protein coronin 1C (CORO1C) in A549 cells. Our study revealed the vital role of miR-206 in suppressing NSCLC metastasis and gave a hint that targeting miR-206 could be a potential therapy for NSCLC metastasis.

## Materials and methods

### Materials

Dulbecco’s Modified Eagle Medium (DMEM), Lipofectamine LTX and PLUS reagents as well as Lipofectamine 3000 transfection reagent were products of Thermo Fisher Scientific (Waltham, MA, USA). 24-well transwell plates (8.0-μm pore polycarbonate membrane inserts) were purchased from Corning (New York, NY, USA). Matrigel was purchased from BD Biosciences (Franklin Lakes, NJ). The pCMV3-CORO1C-WT-GFPSpark vector, pCMV3-CORO1C-Mut-GFPSpark vector (the binding site of miR-206 to CORO1C was replaced with UGCGAGG), NC mimic (5′-UUUGUACUACACAAAAGUACUG-3′) and miR-206 mimic (5′-TGGAATGTAAGGAAGTGTGTGG-3′) were purchased from Sino Biological Inc. (Beijing, China). The E.Z.N.A. Total RNA Kit I was purchased from Omega Bio-Tek (Doraville, GA, USA). The iQ SYBR Green Supermix Kit for q-RT-PCR was a product of Life Technologies (Grand Island, NY, USA). Antibodies against vimentin (D21H3), E-cadherin (24E10), N-cadherin (D4R1H), ZO-1(D6L1E) and Snail (C15D3), CORO1C (D6K5B) and β-actin (13E5) were purchased from Cell Signaling Technology (Danvers, MA). Other reagents used in this study were products of Sigma-Aldrich (St. Louis, MO).

### Patients and tissue samples

We collected 50 pairs of NSCLC tissues and corresponding normal mucosa tissues from the General Hospital of Guangzhou Military Region, Guangzhou, China. Tissues were obtained from the patients who underwent surgery to remove NSCLC tissues in the General Hospital of Southern Theater Command. All patients’ diagnoses were confirmed histopathologically. The study was conducted under the supervision of the General Hospital of Southern Theater Command following the World Medical Association, and all patients have signed informed consent.

### RNA isolation and quantitative reverse-transcription PCR (q-RT-PCR)

Total RNA of the frozen NSCLC tissues and cell lines was extracted using Trizol reagent. The cDNA of mRNA was generated with a Transcriptor First Strand cDNA Synthesis kit, and the cDNA of miRNA was generated with all-in-One miRNA qRT-PCR detection kit. The oligonucleotide primers used to detect miR-206 and CORO1C are presented in Supplementary Material 1.The PCR conditions were as follows: predenaturing at 95 °C for 5 min, then 45 cycles (95 °C for 10 s, 60 °C for 20 s, 72 °C for 20 s), followed by 72 °C for 10 min. All these processes were performed with a Roche LightCycler 480 real-time PCR machine. GAPDH and U6 snRNA were set as endogenous controls for mRNA and miRNA. ΔCt was the result that normalized to U6 or GAPDH Ct values. Then ΔΔCt was calculated by normalizing the ΔCt of the control sample. The values of the target gene are presented as the relative fold change of the housekeeping gene, which was set as 1. Fold change of the gene was calculated by the equation: 2^−ΔΔCt^.

### Cell culture

Human lung cancer cell lines including A549, NCI-H365, NCI-H460, SPCA-1 and XL-1, as well as human lung epithelia cells BEAS-2B, were products of the American Type Culture Collection (ATCC, Manassas, VA, USA). All the cells were cultured with DMEM containing 10% FBS, 100 U/ml penicillin sodium (PS), and 100 mg/ml streptomycin sulfate in a 37 °C incubator containing 5% CO_2_.

### Cell proliferation assay

The 3-(4,5-dimethylthiazol-2-yl)-2,5-diphenyltetrazolium bromide (MTT) assay was used to evaluate cell vitality as previously described [22]. Briefly, after culturing for 24 h, the cells that were seeded in the 96-well plates were transfected with NC mimic or miR-206 mimic for various times (24 h, 48 h, 72 h and 96 h). Then the cell viabilities were measured with MTT assay using a Multi-Detection Microplate Reader (BMG Labtech, Ortenberg, Germany).

### Wound healing assay

Briefly, 5 × 10^5^ A549 cells were seeded in 6 plates and grown to approximately 100% confluence. Then the cells were starved for 6 h with 2% FBS DMEM, followed by scratching using a 10-μl pipette tip. Next, the cells were exposed to NC mimic or miR-206 mimic for an additional 8 h in 2% FBS DMEM. The cells of the same field were photographed at 0 h and 8 h with an Olympus IX70 inverted microscope (Shinjuku, Tokyo, Japan). The Image-Pro Plus 6.0 (IPP 6.0) software (Rockville, MD) was used to calculate the migratory cells and the experiment was carried out in triplicate.

### Transwell migration and transwell invasion assay

We conducted the transwell migration assay and transwell invasion assay to evaluate the effect of miR-206 on the vertical migration ability and invasion ability of A549 cells using a 24-well transwell chamber. The cells suspended in non-serum DMEM were seeded in the upper chamber of the transwell with a density of 2 × 10^4^ per well, and 600 μL of fresh complete DMEM (10% FBS) were added to the lower chamber. The cells were treated with NC mimic and miR-206 mimic at the same time. The cells in the upper chamber were fixed with 4% paraformaldehyde for 30 min after incubating for 24, following by staining with 0.1% crystal violet. After wiping of the non-migratory cells in the inner side of the upper chamber, the migratory cells adhering to the lower surface of the membrane were observed and photographed using an Olympus IX70 inverted microscope. Migratory cells were analyzed by IPP 6.0 software. Regarding the transwell invasion assay, the upper chambers were precoated with Matrigel. Other processes were the same as in the transwell migration assay.

### MiRNA mimic and vector transfection

The miR-206 mimic or NC mimic was transfected into A549 cells using Lipofectamine RNAiMAX transfection reagent (Invitrogen) in the reference of the manufacturer’s instructions.

Regarding vector transfection, the NC vector or CORO1C vector was transfected into adherent A549 cells using Lipofectamine 3000 transfection reagent following the manufacturer’s instructions. Transfected cells were subjected to western blotting assay and q-RT-PCR assay to detect the transfection efficacy.

### Gaussia luciferase reporter assay

Adherent 293 T cells were cotransfected with pCMV3-CORO1C-WT-luc vector/pCMV3-CORO1C-Mut-luc vector (expressing firefly luciferase) and pGL4.73 [hRluc/SV40] vectors (expressing Renilla luciferase) using Lipofectamine LTX and PLUS reagents according to the manufacturer’s protocol. The cells were applied for luciferase signals analyses on a TECAN Infinite F500 platform (Männedorf, Switzerland). The relative luciferase activity was the value that normalized to Renilla luciferase. Three experiments were performed.

### Western blotting assay

We conducted western blotting assay according to a previously described protocol [23] with some modifications. The A549 cells that were transfected with NC mimic or miR-206 mimic for 24 h were collected, lysed with RIPA buffer and subjected to Western blotting assay.

### Tumor xenograft model

5- to 6-week old male BABL/c (nu/nu) mice obtained from Vital River Laboratory Animal Technology Co, Ltd. (Beijing, China) were housed in a specific pathogen-free room. All the animal studies were conducted under the supervision of the General Hospital of Southern Theater Command animal care and use committee. A549 cells suspended in 200 μl of PBS were inoculated subcutaneously into the backs of male mice. After about 10 days, tumor-bearing mice were randomized to the NC mimic group and miR-206 mimic group (five mice per group) and the tail was intravenously injected with NC mimic or miR-206 mimic once every two days for a total of 7 times. A slide caliper was used to measure the tumor volumes, and the formula a × b^2^ × 0.5 (a represents the longest diameter and b is the shortest diameter) was used to calculate the tumor volume. The mice were anesthetized with an intraperitoneal injection of 50 mg/kg pentobarbital sodium. After that, the tumors were removed and weighed. Then the tumors were cryopreserved for further q-RT-PCR experiments. During the experiment, no adverse outcomes occurred, such as blistering or ulceration of tumor.

### Statistical an0061lysis

The data analysis was processed with GraphPad Prism 5.0 (GraphPad Software, Inc., San Diego, CA) and all data were presented as the mean ± standard error of the mean (SEM). Significant differences between two groups were analyzed with two-tailed unpaired Student’s t-test, and the difference of multiple groups was calculated using one-way ANOVA (Tukey’s post hoc test). *P* < 0.05 was identified as statistically significant.

## Results

### MiR-206 expression is decreased in NSCLC tissues and cell lines

**Fig. 1:**
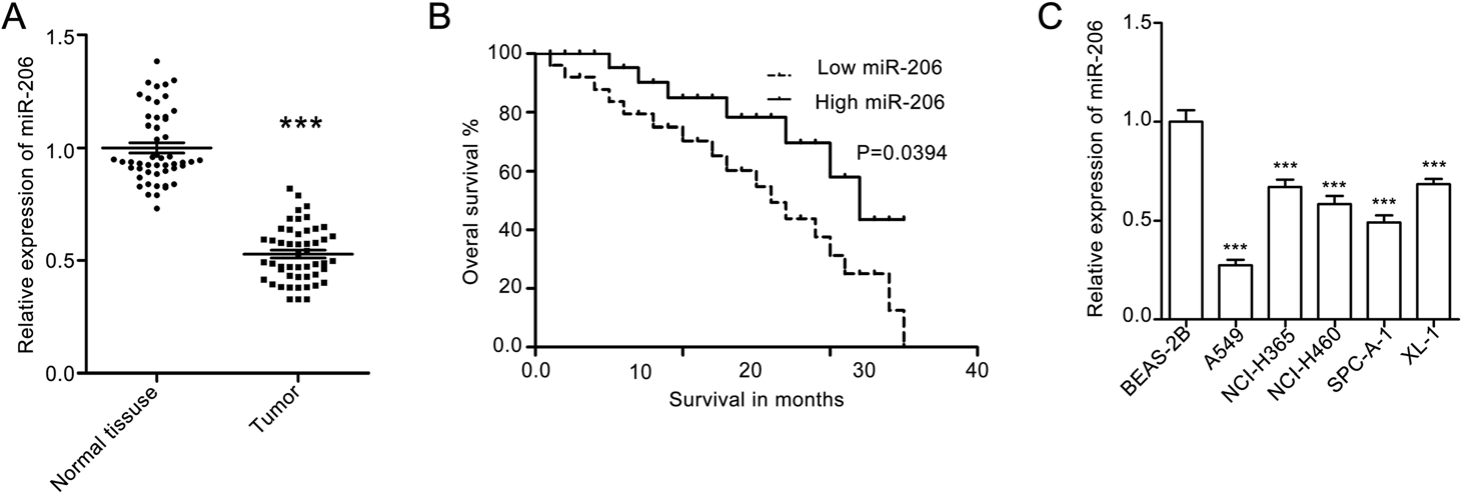
MiR-206 expression is downregulated in NSCLC tissues and cell lines. **a** The miR-206 mRNA level in NSCLC tissues and the corresponding normal mucosa tissues detected by q-RT-PCR assay. Quantitative data are presented as the mean ± SEM, *n* = 50. ***P < 0.001 compared with the normal tissues. **b** Kaplan–Meier curves for overall survival analysis based on miR-206 expression. **c** miR-206 expression in human lung epithelia BEA-2B cells and human lung cell lines, including A549, NCI-H365, NCI-H460, SPCA-1 and XL-1, detected with q-RT-PCR assay. Quantitative data are presented as mean ± SEM, n = 50. ***P < 0.001 compared with BEA-2B cells

To explore miR-206 expression in the NSCLC tissues, we collected 50 paired tissues from the NSCLC patients and detected miR-206 mRNA level using q-RT-PCR. We found that miR-206 expression was much lower than that in adjacent normal mucosa (Fig. 1a). Then we analyzed whether miR-206 expression was correlated with overall survival rate with the Kaplan-Meier method. We found that miR-206 expression was associated with patient survival rate. Patients with low miR-206 expression (the relative miR-206 is lower than the median value of 0.498, *n* = 24) often have poor survival rates compared with patients with high miR-206 expression (the relative miR-206 is greater than the median value of 0.498, *n* = 26) (Fig. 1b). We also detected miR-206 expression in human normal epithelia BEA-2B cells and five lung cancer cell lines (A549, NCI-H365, NCI-H460, SPCA-1 and XL-1). The results showed that miR-206 expression was much lower in lung cancer cells than that in BEA-2B cells, and the miR-206 expression in A549 cells was lowest among the five lung cancer cells (Fig. 1c). All these findings suggest that miR-206 plays an important role in NSCLC.

### MiR-206 inhibits A549 cell proliferation, migration and invasion

**Fig. 2:**
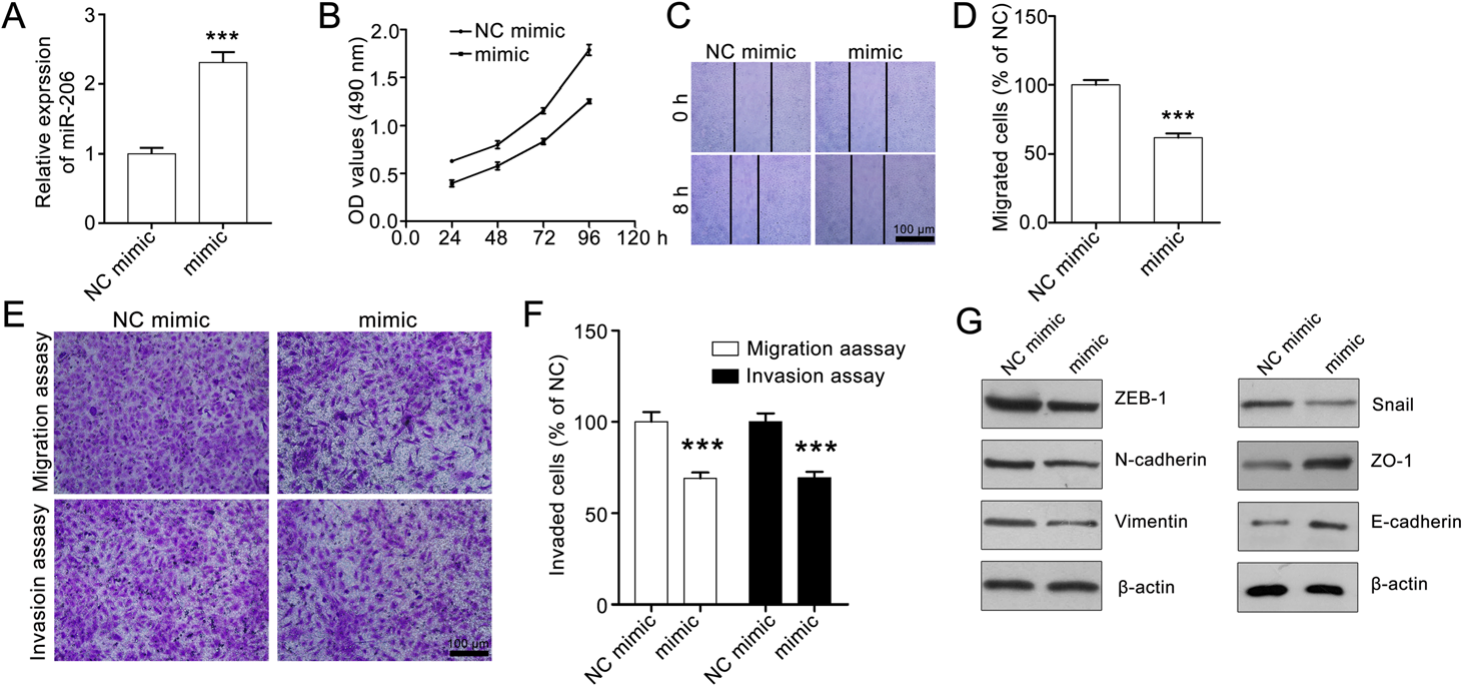
MiR-206 suppresses proliferation, migration and invasion of A549 cells. **a** MiR-206 mimic treatment increased miR-206 mRNA level. **b** MiR-206 represses proliferation of A549 cells. **c** and **d** MiR-206 inhibits horizontal migration of A549 cells. The effect of miR-206 on the horizontal migration of A549 cells was evaluated by wound healing assay. Representative images (100× magnification) and the quantitative data are shown in **c** and **d**, respectively. **e** and **f** MiR-206 represses the migration and invasion of A549 cells. Representative images (100× magnification) of the transwell migration assay and transwell invasion assay are shown in **e**. Quantitative data are shown in **f**. The data were analyzed with GraphPad Prism 5.0 and presented as mean ± SEM, *n* = 3. ***P < 0.001 compared with the control group. **g**. MiR-206 downregulates expression of EMT markers. The A549 cells were treated with miR-206 mimic or NC mimic for 24 h, then the cells were collected and subjected to western blotting assay to detect EMT expression. β-actin was set as a loading control

We next explored whether miR-206 exerts an inhibitory effect on the proliferation, migration and invasion of lung cancer cells. Considering that miR-206 expression in A549 cells was lower than that in other lung cancer cell lines, we selected A549 cells for further assessment in in vitro studies. As shown in Fig. 2a, the miR-206 mimic obviously increased the expression of miR-206 compared with the NC mimic. We treated A549 cells with miR-206 mimic or NC mimic for various times (24–96 h), and then detected cell viability. The results showed that the OD values were reduced in the miR-206 mimic-treated groups compared with those in the NC mimic groups, indicating that miR-206 suppressed A549 cell proliferation (Fig. 2b). Then we conducted wound healing assays to estimate whether miR-206 suppresses the horizontal mobility of A549 cells. We found that after treatment with miR-206 mimic migratory cells were decreased, indicating that miR-206 inhibits the horizontal mobility of A549 cells (Fig. 2c and d). Moreover, we obtained a similar result in the transwell invasion assay. MiR-206 significantly suppressed the invasion abilities of A549 cells (Fig. 2e and f). Also, the western blotting assay showed that miR-206 repressed the biomarkers of EMT (Fig. 2g). Therefore, our study suggests that miR-206 inhibits A549 cell proliferation and metastasis in vitro*.*

### MiR-206 directly targets CORO1C

**Fig. 3:**
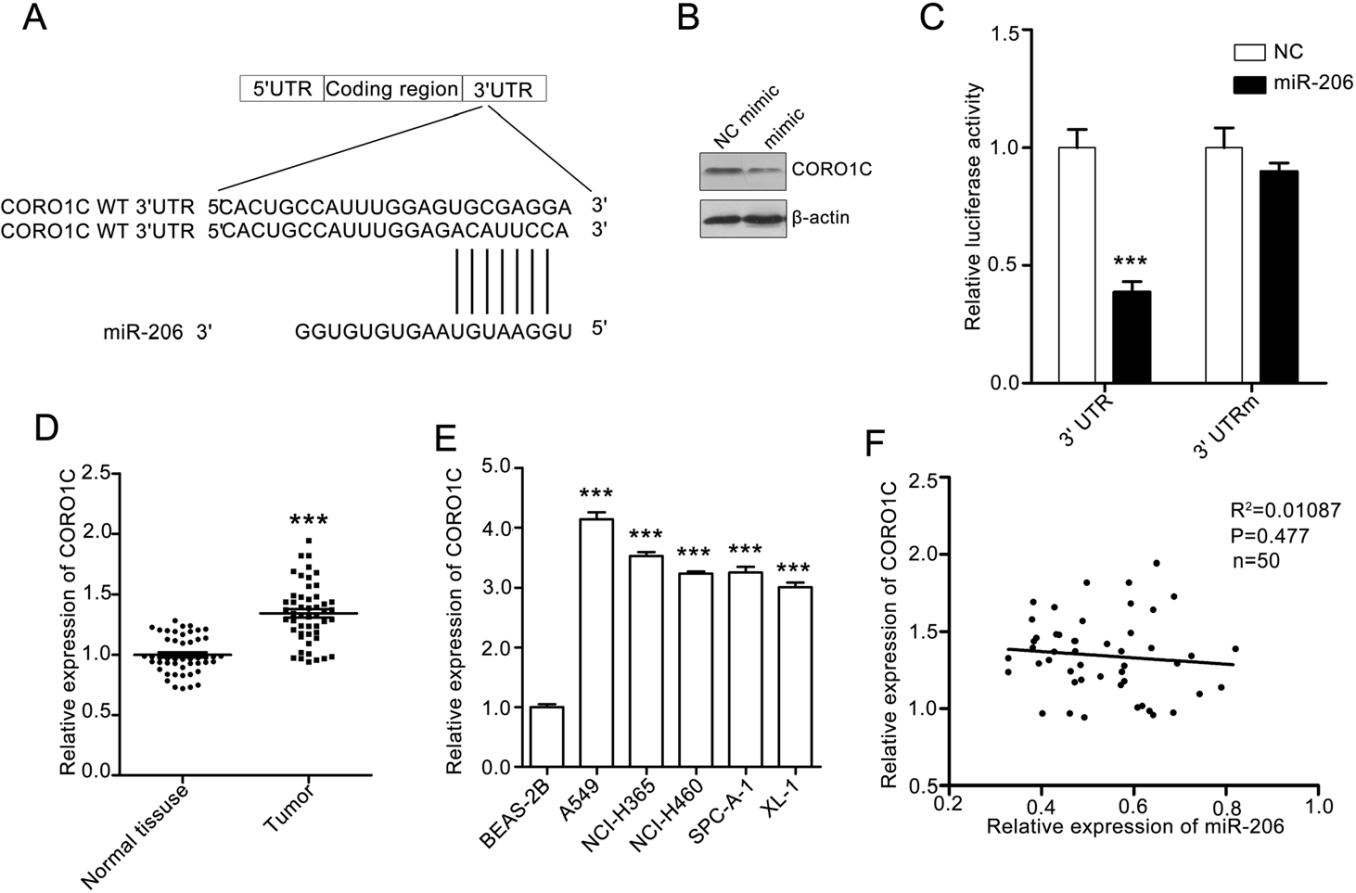
MiR-206 directly targets the 3′-UTR of CORO1C. **a** The sequence of human miR-206 and the predicted binding sites with miR-206 within the CORO1C 3′-UTR are shown. **b** MiR-206 suppresses CORO1C expression in A549 cells. The A549 cells were treated with NC mimic or miR-206 mimic for 24 h, and CORO1C expression was evaluated by western blotting assay. β-actin was used as a loading control. **c** MiR-206 represses CORO1C mRNA in A549 cells. The A549 cells were cotransfected with luciferase plasmids containing wild-type (WT) CORO1C 3′UTR or mutant-type (Mut) CORO1C 3′UTR. The cells were also treated with miR-206 mimic at the same time. The cells were lysed to measure the relative luciferase activity. Quantitative data are presented as mean ± SEM. ***P < 0.001 compared with the NC mimic group. **d** CORO1C expression in NSCLC tissues and corresponding normal mucosa tissues were detected by q-RT-PCR. **e** CORO1C expression in a panel of human lung cell lines and human lung epithelia BEA-2B cells was evaluated by q-RT-PCR. CORO1C expression in BEA-2B was set as 100%. Quantitative data are presented as the mean ± SEM. ***P < 0.001 compared with BEA-2B cells

To explore the underlying mechanism of the miR-206-mediated inhibitory effect on A549 cell behaviors, we searched the TargetScan database to predict the mRNA targets of miR-206. More than 200 mRNAs were predicted to be the downstream target of miR-206. Among them, CORO1C has been demonstrated to play a vital role in tumor metastasis [24, 25]. Therefore, we selected CORO1C for further research (Fig. 3a). Western blotting assay showed that miR-206 stimulation obviously repressed the CORO1C expression (Fig. 3b). To further demonstrate that miR-206 targets CORO1C, we constructed a full-length wild-type CORO1C vector (pCMV3-CORO1C-WT-luc) and 3′-UTR mutant CORO1C vector (pCMV3-CORO1C-mut-luc) of CORO1C. We transfected these two vectors into A549 cells and then conducted a dual-luciferase reporter gene assay. The results showed that the luciferase activities of pCMV3-CORO1C-WT-luc vector were obviously reduced after miR-206 treatment whereas the luciferase activities of pCMV3-CORO1C-WT-luc vector was almost unchanged (Fig. 3c). These results demonstrated that miR-206 directly targets CORO1C.

Next, a q-RT-PCR assay was performed to detect the CORO1C expression in NSCLC tissues and adjacent normal tissues. We found that the CORO1C expression increased in NSCLC tissues (Fig. 3d). In addition, we detected CORO1C expression in BEAS-2B cells and the five lung cancer cell lines. Similar to the q-RT-PCR result in tissues, CORO1C was upregulated in the five lung cancer cell lines compared with BEAS-2B cells (Fig. 3e). We also analyzed the correlation of miR-206 expression and CORO1C expression. The patients with low miR-206 expression tended to express a high CORO1C level, but there was no significant correlation between miR-206 expression and CORO1C expression (Fig. 3f). Thus, all these results indicate that miR-206 negatively regulates tumor metastasis by directly targeting CORO1C.

### CORO1C overexpression rescues miR-206-mediated inhibition of A549 cells

**Fig. 4:**
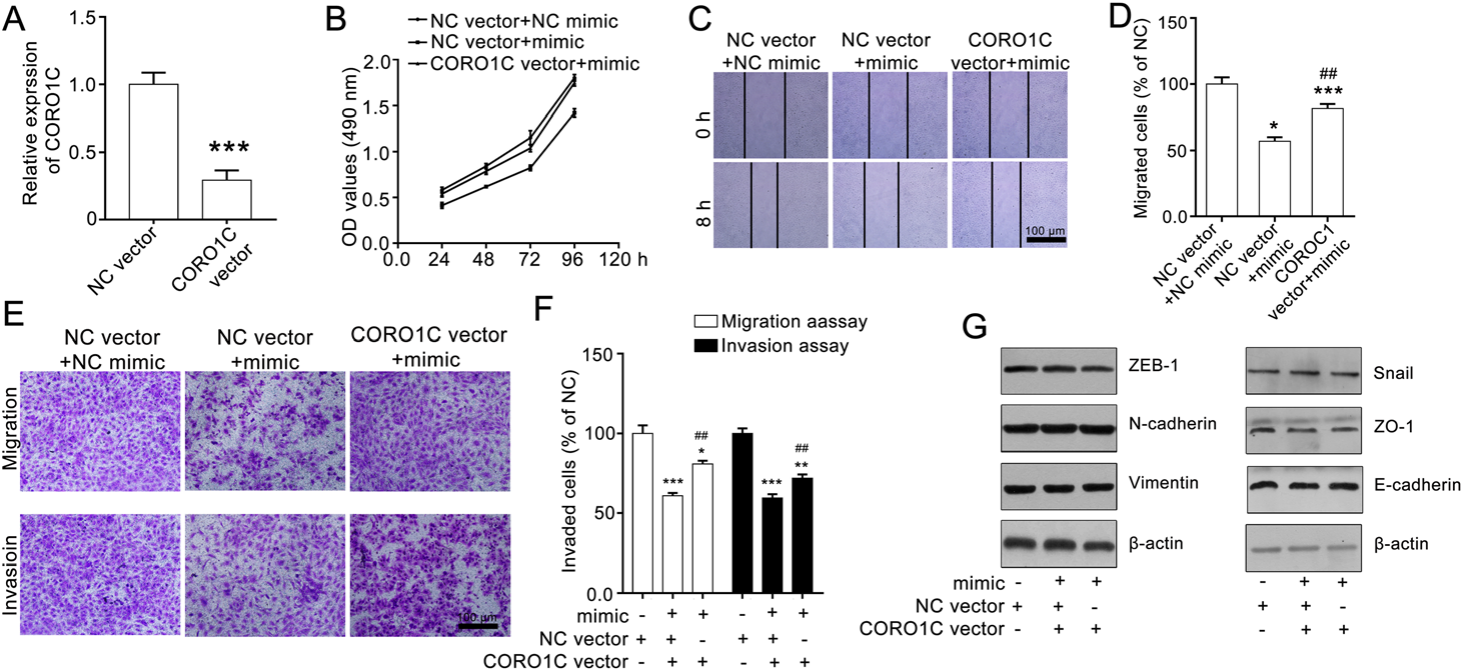
CORO1C overexpression attenuates miR-206-mediated inhibitory effect on A549 cells. **a** CORO1C vector transfection increased CORO1C expression. The cells were treated with CORO1C vector or NC vector for 24 h, then the cells were collected for q-RT-PCR assay. **b** CORO1C vector transfection decreases miR-206-mediated inhibition effect on A549 cell proliferation. The cells were transfected with NC vector and CORO1C vector, and then treated with miR-206 mimic for 24 h. Then the cells were subjected to MTT assay. **c** and **d**) CORO1C overexpression rescues miR-206-induced effect on the horizontal migration of A549 cells. A549 cells were transfected with NC vector or CORO1C vector, and then applied for wound healing assay stimulated with miR-206 mimic. Representative images (100× magnification) and the quantitative data are shown in **c** and **d**, respectively. (**e** and **f**) CORO1C vector attenuates miR-206-mediated inhibitory effect on the vertical migration and invasion of A549 cells. A549 cells were transfected with NC vector or CORO1C vector, and then subjected to transwell migration and transwell invasion assays. Representative images (100 × magnification) and the quantitative data are shown in **e** and **f**, representatively. **g** CORO1C vector rescues miR-206-induced inhibitory effect of EMT markers. The cells were transfected with CORO1C vector or NC vector. After 24 h the cells were collected and subjected to western blotting assay. Quantitative data are presented as mean ± SEM. *P < 0.05 and ***P < 0.001 compared with the NC vector group, ^#^*P* < 0.05 and ^##^*P* < 0.01 compared with the NC vector+ NC mimic group

To further verify that miR-206 inhibited the migration and invasion of A549 cells via negatively targeting CORO1C, we transfected A549 cells with CORO1C vector or NC vector and showed that CORO1C vector transfection significantly up-regulated CORO1C expression (Fig. 4a). Then we evaluated the effect of miR-206 on the transfected cells. As shown in Fig. 4b, after transfecting CORO1C vector, miR-206 had no significant inhibitory effect on A549 cells, and CORO1C overexpression rescued miR-206-induced inhibitory action on A549 cells’ horizontal migration ability (Fig. 4c and d). We also observed the same result in the transwell migration and transwell invasion assays. CORO1C vector transfection obviously reversed the miR-206-induced inhibitory effect on A549 cell invasion (Fig. 4e and f). In addition, CORO1C deletion rescued the miR-206-mediated downregulation of EMT biomarkers (Fig. 4g). Thus, miR-206 negatively regulates the proliferation, migration and invasion of A549 cells via targeting CORO1C.

### MiR-206 suppresses the tumor growth and EMT marker in A549 xenografts

**Fig. 5:**
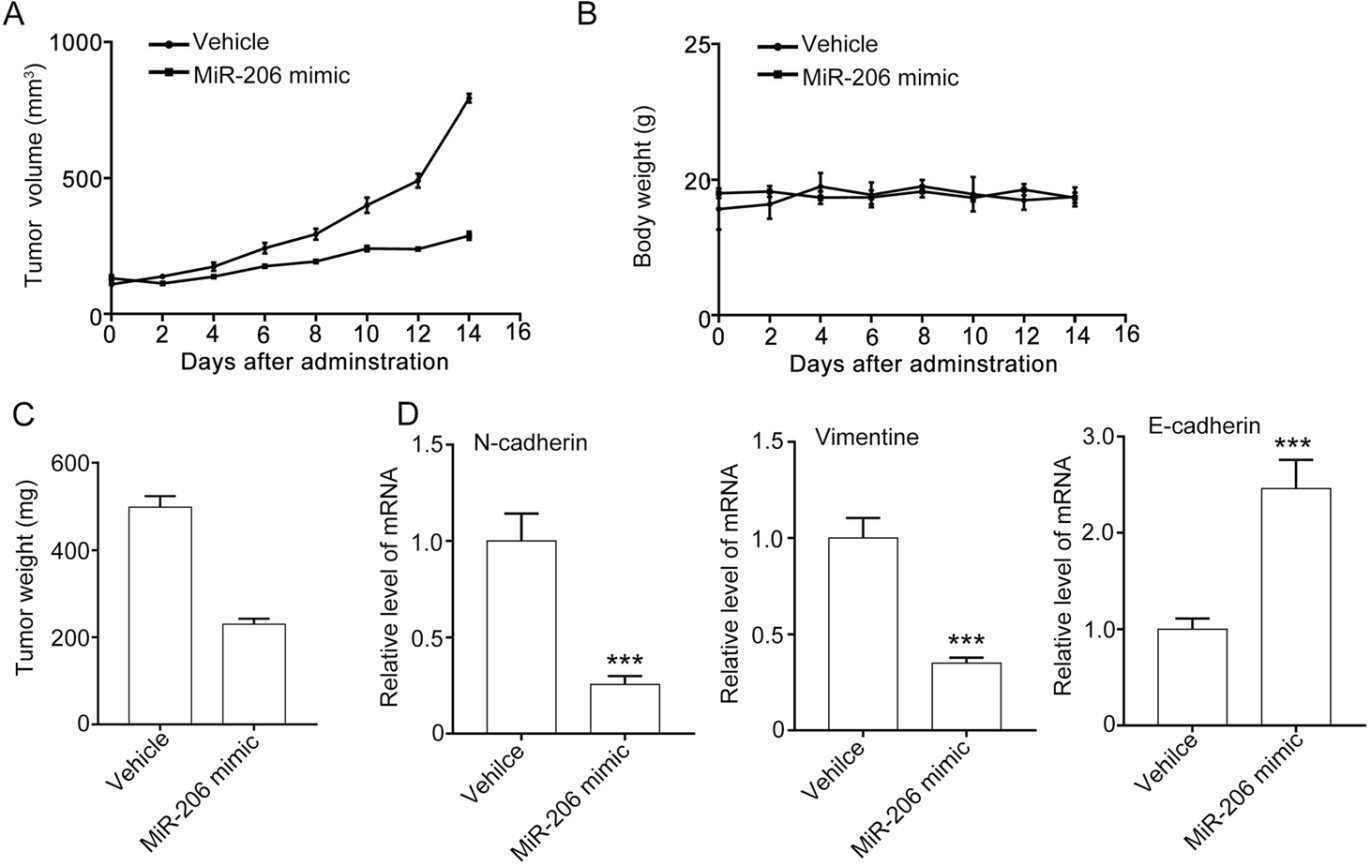
MiR-206 suppresses tumor growth of A549 xenograft mouse model. (**a** and **b**) MiR-206 inhibited A549 xenograft tumor growth. A549 cells suspended in PBS were injected subcutaneously into 5- to 6-week-old BABL/c (nu/nu) male mice to establish the xenograft. Then the mice were divided into the NC mimic group and miR-206 mimic group. The tumor volumes were measured by a slide caliper. At the end of the experiment, tumors were removed and frozen. The tumor volume curve and tumor weight are shown in **a** and **b**, respectively. **c** MiR-206 suppressed mRNA of EMT marker in tumor. Quantitative data are presented as mean ± SEM. ***P < 0.001 compared with the NC mimic group

We further explored the function of miR-206 in a A549 xenograft mouse model. The results showed that miR-206 treatment significantly suppressed the tumor growth, indicated by the tumor volumes (about 300 mm^3^) and tumor weight in the miR-206 mimic being lower than those in the NC mimic group (about 800 mm^3^) (Fig. 5a and b). We also found that miR-206 down-regulated the mRNA of N-cadherin and vimentin, and up-regulated the mRNA level of E-cadherin, suggesting that miR-206 can suppress the EMT of A549 xenografts (Fig. 5c). In conclusion, miR-206 inhibits the proliferation, migration and invasion in vitro and in vivo.

## Discussion

NSCLC is the major cause of cancer-related mortality. Metastasis is considered as one of the major factors leading to NSCLC mortality. Various miRNAs have been shown to be vital regulators of NSCLC metastasis. However, the role of miR-206 in NSCLC metastasis and the underlying mechanism remain unclear [20, 21]. In the present study, we demonstrated that miR-206 is a NSCLC metastasis suppressor, and low miR-206 expression often suggests a poor survival rate. We also demonstrated that miR-206 inhibited cell proliferation, migration and invasion in A549 cells via negatively regulating CORO1C. This study indicates that miR-206 is a potential target for NSCLC therapy.

NSCLC is the dominant cause of the cancer-related death worldwide [26, 27]. Although many efforts have been made for NSCLC therapy, the results are limited and unsatisfactory. Metastasis is one of the most important factors responsible for the therapy failure [2, 28]. Identifying the molecular genetics involved in NSCLC metastasis is beneficial for understanding the mechanism and contributing to NSCLC therapy [1]. Numerous studies have demonstrated that miRNAs are vital for NSCLC metastasis and that targeting miRNAs may be an effective therapeutic strategy for NSCLC [13, 29]. For example, miR-203 functions as a tumor metastasis suppressor through inactivating the ERBB4/PIK3R3/mTOR/S6K2 signaling pathway, and miR-203 overexpression inhibited NSCLC metastasis in vitro and in vivo [15]. MiR-135b promotes NSCLC metastasis by regulating multiple targets in the Hippo pathway and LZTS1. Targeting miR-135b is a potential therapeutic strategy for NSCLC [16]. All these observations showed that miRNA is a potential therapeutic target of NSCLC. In this study, we demonstrated that miR-206 is vital for NSCLC metastasis. Further studies showed that miR-206 acted as a metastasis suppressor by negatively targeting CORO1C. In this regard, our study contributes to the understanding of the molecular genetics for NSCLC and provides evidence suggesting that miR-206 is a potential target of NSCLC therapy. Our study also provides new insight into the underlying mechanism of miRNAs in NSCLC metastasis.

MiR-206 is one of the best characterized miRNAs and is specifically expressed in skeletal muscle [21]. It functions as a tumor metastasis suppressor in a series of cancers, including colon cancer, renal cell carcinoma, breast cancer and gastric cancers [17–19]. However, its role, molecular mechanism and clinical significance in NSCLC are still not completely understood. In the current study, we reported that miR-206 expression was reduced in NSCLC, and low miR-206 expression often suggests poor survival. MiR-206 suppressed A549 cell metastasis in vitro via targeting CORO1C. Our study reveals the role of miR-206 in NSCLC metastasis and suggests that miR-206 expression may be a biomarker for calculating the survival rate during NSCLC therapy.

CORO1C is a WD-repeat protein and plays a vital role in actin-dependent processes. CORO1C is one of the most important factors responsible for controlling and remodeling of actin filament networks. Numerous studies have shown that CORO1C is vital for cell motility by regulating cell motility via actin filament turnover coordination. CORO1C is overexpressed in multiple types of clinically aggressive cancers, such as triple-negative breast cancer, gastric cancer and glioblastoma. CORO1C deletion significantly represses cell invasion and metastasis [30–32]. In addition, CORO1C has been identified as the target of many molecular factors, such as miR-133a, miR-206 and Y-box binding protein-1 (YBX-1) [24, 25, 32]. However, its role in NSCLC metastasis is still unknown. In this study, we showed that CORO1C was upregulated in NSCLC tissues as well as a series of lung cancer cell lines. We also showed that miR-206 was an important factor responsible for the regulation of CORO1C in NSCLC. CORO1C overexpression can obviously attenuate miR-206-mediated inhibitory effects on the proliferation, migration and invasion of A549 cells, indicating that CORO1C is important for NSCLC metastasis. Thus, our study provides strong evidence for the role of CORO1C in NSCLC metastasis and sheds new light on the clinical significance of CORO1C in cancer therapy. Regarding the lack of a significant correlation between miR-206 expression and CORO1C expression, there are two possible reasons for this result. Firstly, the underlying mechanism of the miR-206-mediated inhibitory effect on NSCLC is complicated. Besides CORO1C, miR-206 may inhibit NSCLC proliferation, migration, and invasion via other targets, partly indicating that there is no significant correlation between CORO1C expression and patient survival (Supplementary Material 1). Secondly, we analyzed miR-206 e and CORO1 correlation using 50 pairs of NSCLC tissues; the sample size was underpowered to show statistical differences of the correlation assay. We will further explore the role of miR-206 and CORO1C in the future.

Sry-related high-mobility group (HMG) box 9 (SOX9) is critical for development, differentiation and lineage commitment of various tissues. SOX9 also plays an important role in cancer, and SOX9 inhibition remarkably suppressed the proliferation, migration and invasion of NSCLC cells. In addition, SOX9 overexpression increased cell migration and invasion and significantly promoted EMT (33, 34). MiR206 inhibits the proliferation, migration and invasion of NSCLC, partially via targeting SOX9 (35). Both SOX9 and CORO1C are downregulated targets of miR-206. As a transcription factor, SOX9 may regulate CORO1C expression, and we will investigate the relationship between SOX9 and CORO1C in future studies.

In conclusion, our study demonstrated that miR-206 is an NSCLC metastasis suppressor. Mechanism studies demonstrated that miR-206 inhibited the proliferation, migration and invasion of A549 cells via negatively targeting CORO1C. This study provides evidence that miR-206 functions as a suppressor of NSCLC metastasis by negatively regulating CORO1C and indicates that targeting miR-206 may be a potential therapeutic strategy for NSCLC.

## Supplementary information

**Additional file 1.** Supplementary Material 1. Primers used in this study.

## Data Availability

All data generated or analyzed during this study are included in this published article.

## Abbreviations

3′-UTR: 3′-untranslated region
CORO1C: Coronin-1C
DMEM: Dulbecco’s Modified Eagle Medium
EMT: Epithelial-mesenchymal transition
FBS: Fetal bovine serum
miR-206: Micro-206
mRNA: Messenger RNAs
MTT: 3-(4,5-dimethylthiazol-2-yl)-2,5-diphenyltetrazolium bromide
NSCLC: Non-small-cell lung cancer
q-RT-PCR: Quantitative reverse-transcription PCR
