# Supplementary material for: MiR-206 may suppress non-small lung cancer metastasis by targeting CORO1C
Source: Cell Mol Biol Lett. 2020 Mar 17;25:22. doi: 10.1186/s11658-020-00216-x (PMC7079403; doi:10.1186/s11658-020-00216-x)
Supplement: Supplementary file 1 — Additional file 1. Supplementary Material 1. Primers used in this study. [file 11658_2020_216_MOESM1_ESM.docx]

Supplementary Material 1. Primers used in this study.

| Primers | Sequences |
| --- | --- |
| miR-206 F | 5ʹ-GGAATGTAAGGAAGTGTG-3ʹ |
| MiR-206 R | 5ʹ-GAGCAGGCTGGAGAA-3ʹ |
| U6 F | 5ʹ-CTCGCTTCGGCAGCACA-3ʹ |
| U6 R | 5ʹ-AACGCTTCACGAATTTGCGT-3ʹ |
| CORO1C F | 5ʹ-TCCTCCCTCTGCACAAGACT-3 |
| CORO1C R | 5ʹ-GGATCTGCCATACCATGACC 3ʹ |
| Vimentin F | 5ʹ-GCAGGAGGCAGAAGAATGGTA-3ʹ |
| Vimentin R | 5ʹ-GGGACTCATTGGTTCCTTTAAGG-3ʹ |
| E-cadherin F | 5ʹ-CAGGTCTCCTCATGGCTTTGC-3ʹ |
| E-cadherin R | 5ʹ-CTTCCGAAAAGAAGGCTGTCC-3ʹ |
| N-cadherin F | 5ʹ-AGCGCAGTCTTACCGAAGG-3ʹ |
| N-cadherin R | 5ʹ-TCGCTGCTTTCATACTGAACTTT-3ʹ |
| GAPDH F | 5′-GTGAACCATGAGAAGTATG-3′ |
| GAPDH R | 5′-CGGCCATCACGCCACAGTTTC-3′ |


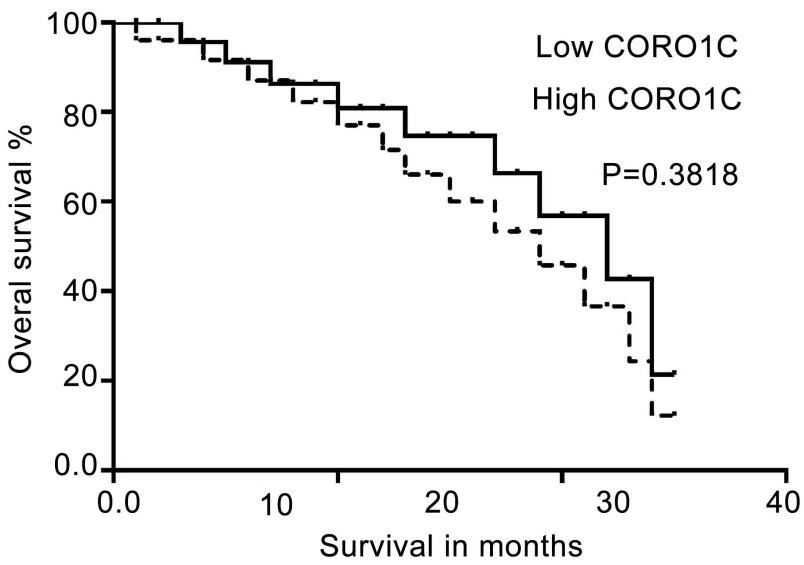


Supplementary Material 2. Kaplan–Meier curves for overall survival analysis based on CORO1C expression.


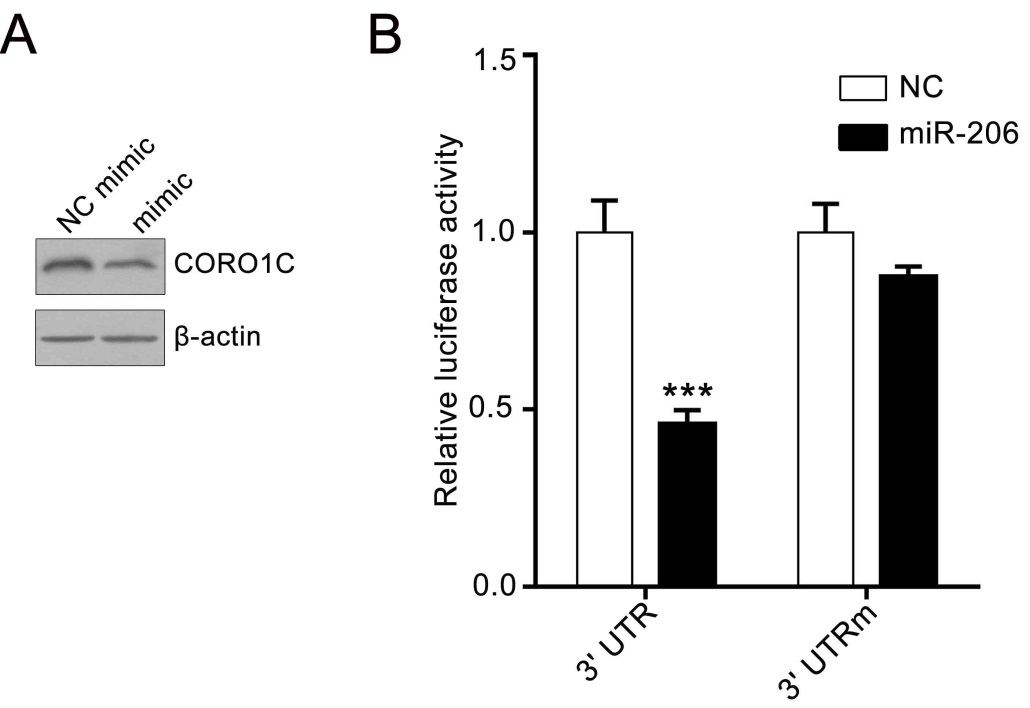


Supplementary Material 3. MiR-206 suppresses CORO1C expression in SPCA-1 cells. SPCA-1 cells were treated with NC mimic or miR-206 mimic for 24 h, and CORO1C expression was evaluated by western blotting assay. β-actin was used as a loading control. **(C)** MiR-206 represses CORO1C mRNA in SPCA-1 cells. The A549 cells were cotransfected with luciferase plasmids containing wild-type (WT) CORO1C 3ʹUTR or mutant-type (Mut) CORO1C 3′UTR. The cells were also treated with miR-206 mimic at the same time. The cells were lysed to measure the relative luciferase activity. Quantitative data are presented as the mean ± SEM. ****P* < 0.001 compared with the NC mimic group.
